# Self-assembled organic nanorods for dual chemo-photodynamic therapies†

**DOI:** 10.1039/c8ra00067k

**Published:** 2018-02-01

**Authors:** Yuanyuan Li, Xiuli Hu, Xiaohua Zheng, Yang Liu, Shi Liu, Ying Yue, Zhigang Xie

**Affiliations:** The First Hospital of Jilin University Xinmin Street Changchun Jilin 130021 PR China yying119@126.com; State Key Laboratory of Polymer Physics and Chemistry, Changchun Institute of Applied Chemistry, Chinese Academy of Sciences Changchun 130022 China lily@ciac.ac.cn; University of Science and Technology of China Hefei 230026 PR China; Department of Chemistry, Northeast Normal University 5268 Renmin Street Changchun 130024 P. R. China

## Abstract

Photodynamic therapy (PDT) and chemotherapy have been extensively developed as effective approaches against cancer. Herein, we constructed organic nanorods by rational co-assembly of photosensitizer, di-iodinated borondipyrromethene (BDP-I_2_), and chemical anticancer drug, paclitaxel (PTX). The physico- and photochemical properties of the obtained nanorods were carefully investigated. BDP-I_2_ was selected for its high singlet oxygen (^1^O_2_) quantum yields. And the corresponding ^1^O_2_ generation ability and photodynamic effect were evaluated both *in vitro* and *in vivo*. The accelerated endosomal escape of the nanorods induced by the photodynamic effect enhanced the chemotherapeutic efficacy of PTX. We believe that this synergetic nanomedicine represents a new development for antitumor chemophotodynamic therapy.

## Abstract 2

Organic nanorods prepared by co-assembly of photosensitizer (BDP-I_2_) and chemical anticancer drug, paclitaxel (PTX) were evaluated for chemo-photodynamic therapies.

## Graphical abstract

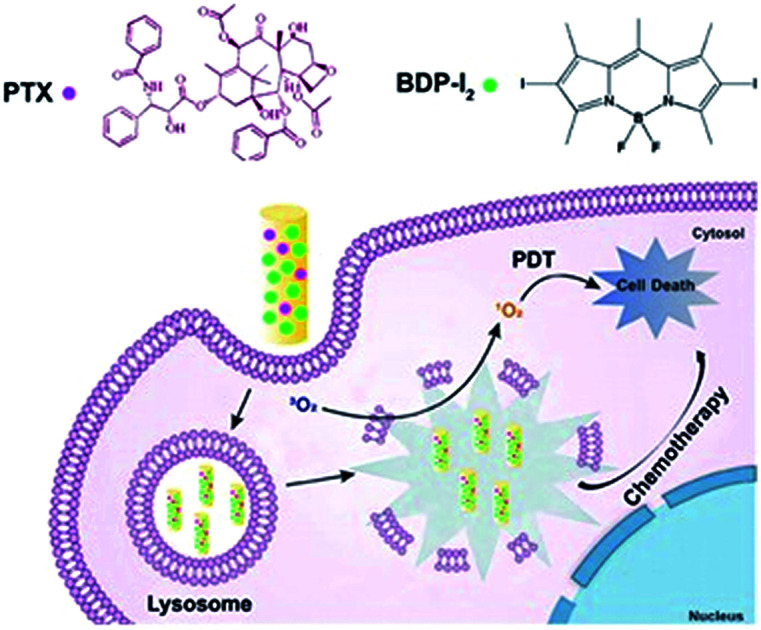

## Introduction

The high fatality rates of cancer have prompted rapid development of various treatments, including radiotherapy, chemotherapy, surgery and phototherapy.^1,2^ Among which, photodynamic therapy (PDT), which can transfer energy from light to the surrounding molecular oxygen (^3^O_2_) and produce cytotoxic reactive oxygen species (ROS), especially singlet oxygen (^1^O_2_) that can lead to an increase in cell membrane permeability and further destroy the cells by disturbing their normal function, has been widely utilized as a noninvasive approach for cancer therapy.^3–8^ The concepts of combining chemotherapy with radiotherapy, or PDT have been developed and proved to be efficient approaches to maximize the treatment outcome while minimize the side effects of each therapy.^5,6,9–13^ For example, Zhou and co-workers have designed a step-by-step multiple stimuli-responsive nanoplatform for enhancing combined chemo-photodynamic therapy.^6^ Gu and co-workers utilize light-activated-responsive nanocarriers for enhanced anticancer therapy.^5^ However, the current delivery systems are mostly based on loading photosensitizer or chemotherapeutic drugs into one or different carriers. The drug loading contents and ratios are uncontrollable. Furthermore, the carrier-related side toxicities also remain concerning.^14^ It is high challenging to develop nanoplatforms that combine chemotherapy and PDT with controlled drug ratios and limited excipients.

Borondipyrromethene (BODIPY) and its derivatives have received much attention and been considered as potential candidates as both bioimaging and PDT therapeutic agents owing to their excellent photostability, high molar extinction coefficient and quantum yields.^14–20^ Halogen atoms are commonly introduced for their efficient triplet-sensitization to BODIPY.^15^

**Scheme 1:**
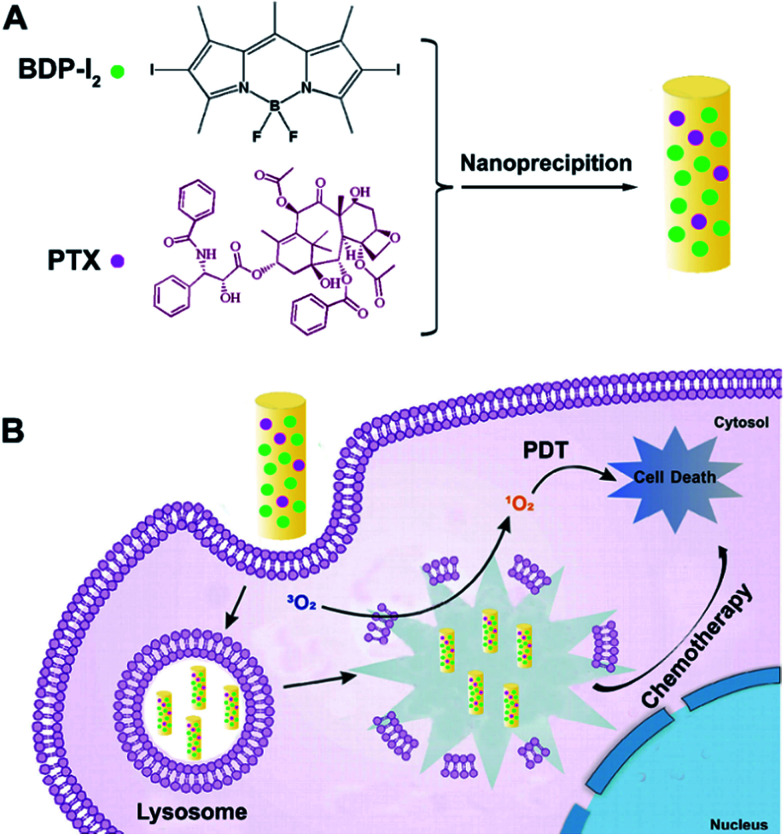
Schematic illustration of the light-activated Co-NRs. (A) Schematic design of Co-NRs. (B) Photodynamic therapy of Co-NRs.

Direct assembly of organic molecules has been proved to be a facile and useful method to obtain nanoplatforms with high drug loading and precise specific molecular structure.^21–25^ Morphology plays an important role in the performance of nanomaterials. Nanoparticles with different size and surface charge have been widely investigated. In our previous study, rod-shaped particles appear to be more favorably engulfed by cells compared to spherical counterparts.^16^ Herein, we construct nanorods (Co-NRs) by the co-assembly of BDP-I_2_ and paclitaxel (PTX) as shown in Scheme 1. The synthesis, characterization, and cellular behavior of the obtained Co-NRs are investigated. And the *in vivo* and *in vitro* photodynamic and chemotherapy effects are also evaluated.

## Results and discussion

**Fig. 1:**
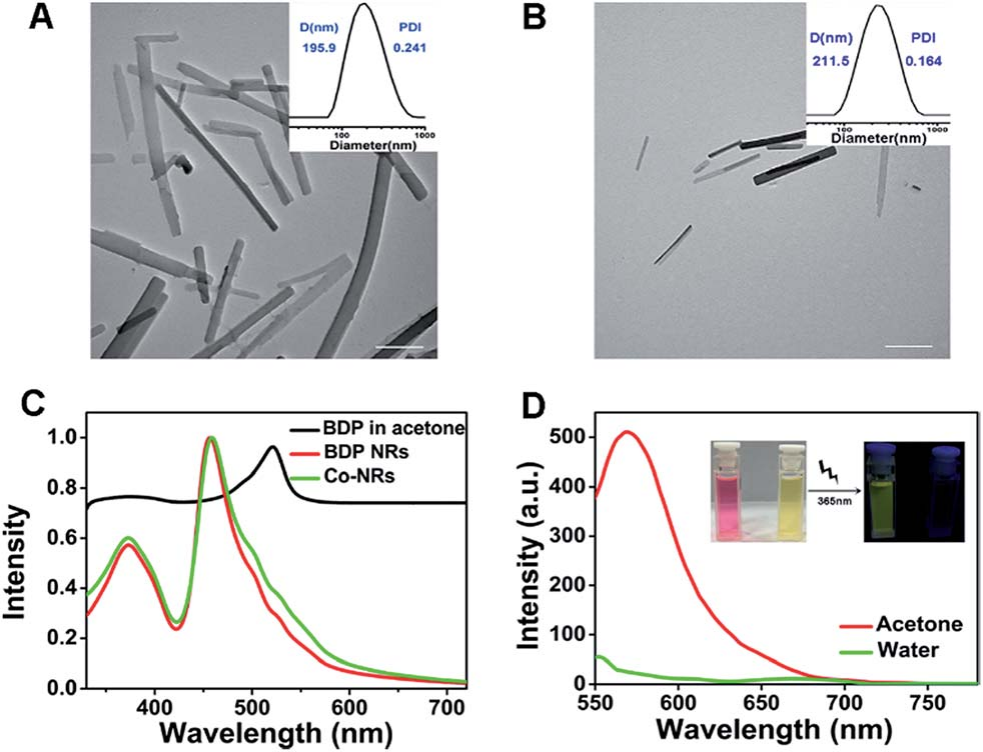
The characterization of BDP-I_2_ NRs and Co-NRs. TEM images and DLS results of (A) BDP-I_2_ NRs and (B) Co-NRs. Scale bars, 500 nm. (C) Normalized UV-vis absorption of BDP-I_2_ in acetone, BDP-I_2_ NRs and Co-NRs in water. (D) Fluorescence spectra of BDP-I_2_ in acetone and BDP-I_2_ NRs in water. Insets: photographs under UV light.

BDP-I_2_ was synthesized according to the previously reported method.^26^ The ^1^H NMR spectroscopy and MALDI-TOF mass spectrum confirmed its accurate chemical structure (Fig. S1, ESI†). BDP-I_2_ could self-assemble or co-assemble with PTX into nanorods (NRs). The concentration of BDP-I_2_ and PTX in Co-NRs were determined to be 10 μg mL^−1^ and 2 μg mL^−1^, respectively, by UV-vis standard curve and high performance liquid chromatography (HPLC) (Fig. S2, ESI†). Transmission electron microscopy (TEM) images confirmed that both BDP-I_2_ NRs and Co-NRs possessed rod morphology with a width of less than 200 nm. Meanwhile, dynamic light scattering (DLS) profiles identified that the corresponding hydrodynamic diameters of BDP-I_2_ NRs and Co-NRs were 195.8 and 211.5 nm, respectively (Fig. 1A–B). Moreover, the size and size distribution of Co-NRs in water almost kept unchanged up to two weeks at room temperature, and kept unchanged for 7 days in 10% PBS-containing water solution or 10% fetal bovine serum (FBS) containing water solution, indicating the good stability of as-prepared Co-NRs in physiological conditions (Fig. S3A–C, ESI†). The zeta potential of Co-NRs also showed not obvious fluctuation after two weeks (Fig. S3D, ESI†). All these results demonstrated the favorable physiological properties of Co-NRs.

Following, the photophysical properties of Co-NRs and BDP-I_2_ were tested. UV-vis absorption spectra revealed that the maximum absorption peak of BDP-I_2_ changed from 521 nm in acetone to 459 nm in NR state (Fig. 1C) due to its aggregation into nano-rods. The Co-NRs and BDP-I_2_ NRs showed almost the same UV-vis absorbance, indicating that adding PTX did not influence the absorbance of BDP-I_2_ NRs. The fluorescence peak of BDP-I_2_ small molecule in acetone was 569 nm excited at 521 nm. However, Co-NRs exhibited almost no fluorescence due to the aggregation-induced quenching (Fig. 1D). The inserted mage in Fig. 1D exhibited the photographs of the florescence difference excited at 365 nm.

**Fig. 2:**
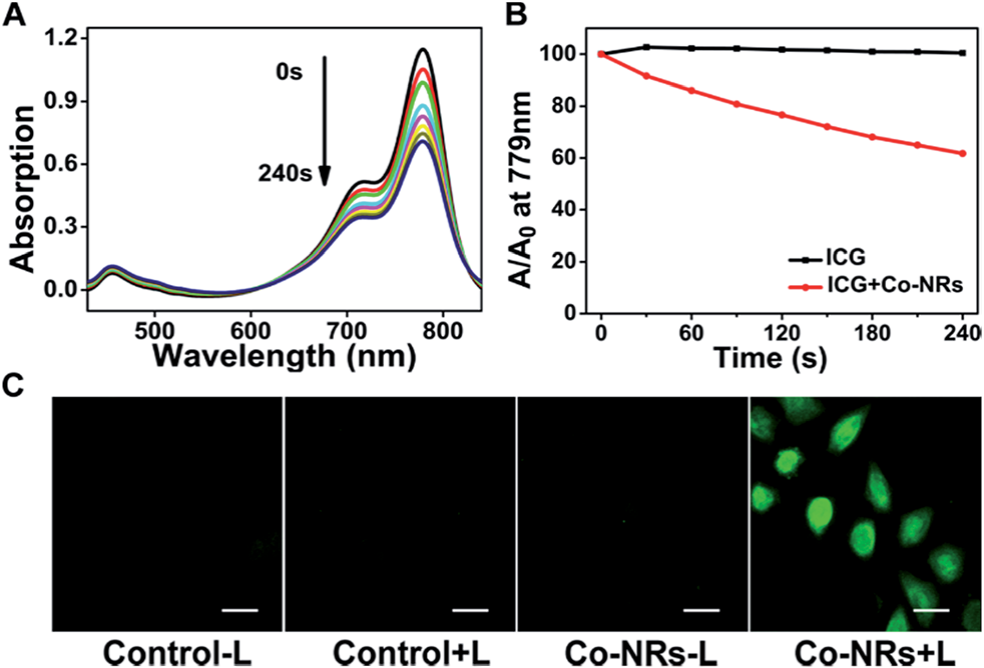
The ROS generation ability of the Co-NRs. (A) Time-dependent UV-vis absorption spectra of ICG at 779 nm upon irradiation with a 540 nm LED light from 0 to 240 s. (B) Comparison of decay rates of ICG alone and blending of ICG and Co-NRs upon irradiation. (C) Generation of ROS *in vitro* upon irradiation (540 nm, 13 mW cm^−2^, 20 min) indicated by the fluorescence of DCF. Scale bars, 20 μm.

Indocyanine Green (ICG) and 2′,7′-dichlorodihydrofluorescein diacetate (DCFH-DA) were used as the indicators to investigate the reactive oxygen species (ROS) generation ability monitored by UV-vis absorption spectra. As shown in Fig. 2A–B, the maximum absorption of ICG at 778 nm showed a time-dependent decrease upon irradiation due to oxidation of ICG by ROS produced by BDP-I_2_. In contrast, ICG alone was stable under light irradiation, indicating the generation of ROS by BDP-I_2_ in Co-NRs. As the photostability of photosensitizers (PSs) was very important for the long time application in biology,^27–30^ the photostability of BDP-I_2_ in Co-NRs was also tested. As shown in Fig. S4 (ESI†), the absorption of Co-NRs almost kept unchanged under irradiation with a 540 nm lamp at 13 mW cm^−2^ for 20 min, indicating its excellent photostability.

Besides, the intracellular ROS generation by Co-NRs was further tested in cervical cancer cells (HeLa cells) by using confocal laser scanning microscopy (CLSM). As shown in Fig. 2C, almost no green fluorescence appeared in the three control groups, indicating there was no adequate ROS generation in cells to oxidize no-fluorescent DCFH-DA to fluorescent dichlorofluorescein (DCF).^31–38^ In contrast, bright green fluorescence emerged in Co-NRs group with light irradiation, suggesting the generation of ROS.

The cellular uptake of Co-NRs by HeLa cells was studied by CLSM. The cellular nuclei were stained with DAPI to blue. With the time prolonged from 0.5 h to 6 h, enhanced fluorescence was observed (Fig. S5, ESI†). The fluorescence of Co-NRs decreased obviously when the incubation temperature changed from 37 °C to 4 °C, indicating the ATP-dependent endocytosis pathway^39–42^ (Fig. S5, ESI†). With light irradiation, a brighter fluorescence appeared for Co-NRs than the control group, suggesting the enhanced cellular uptake due to the photochemical internalization (PCI) effect.^13,43–48^ (Fig. S5, ESI†).

**Fig. 3:**
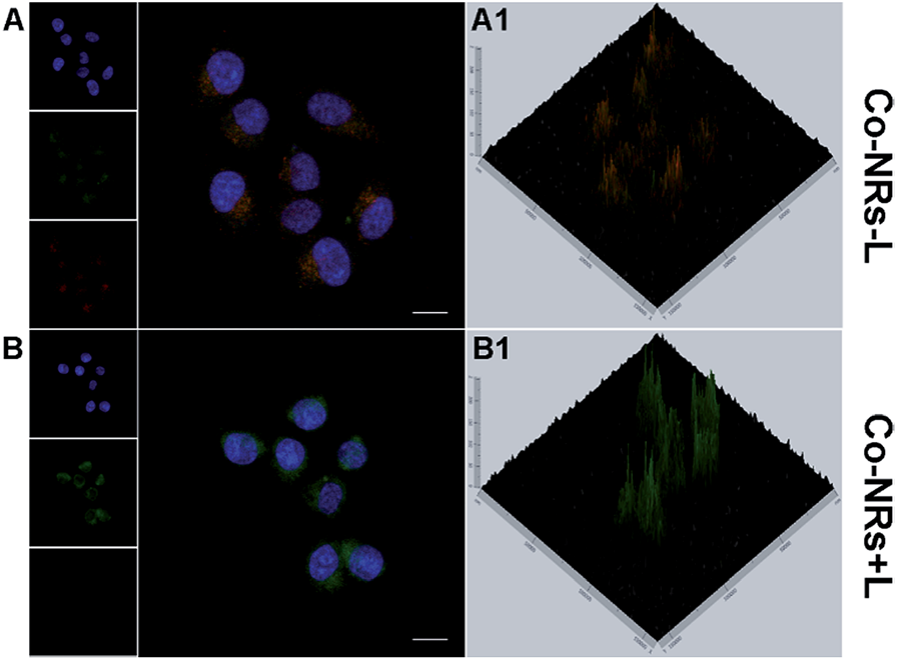
CLSM images of HeLa cells incubated with Co-NRs in the presence of Lyso-tracker Red without (−L) or with (+L) light irradiation. (A1–B1) Fluorescence of Co-NRs and Lyso-tracker Red in cells at 2.5D mode. For each panel, the images from above to down show cell nuclei stained by DAPI (blue), fluorescence of Co-NRs (green), lysosomes stained with Lyso-tracker Red (red) and overlays of three images. Scale bars, 20 μm.

Lyso-tracker Red was employed as a probe to confirm the lysosome colocalization and escape of Co-NRs from lysosome.^49–56^ As shown in Fig. 3A, after incubation with Co-NRs for 2 h, HeLa cells were stained with Lyso-tracker Red at 37 °C for 30 min. The red fluorescence co-localized well with the green fluorescence. After irradiation, enhanced green fluorescence and decreased red fluorescence were observed, confirming that the light irradiation facilitated the escape of Co-NRs from lysosome to cytoplasm due to the photochemical rupture of lysosome membranes (Fig. 3A–B). Lysosome escape further guaranteed the chemotherapeutic effect of PTX in Co-NRs.

Additionally, Acridine Orange (AO) staining test further confirmed the lysosome escape.^57–60^ In lysosomes, the concentrated AO emitted a granular red fluorescence, and a diffuse green fluorescence in cytosol. With the decreased red fluorescence and the increased green fluorescence upon light irradiation, the relocation of AO from the lysosomes to the cytosol occurred, indicating the change of lysosome permeability due to the generated ROS (Fig. S6, ESI†).

**Fig. 4:**
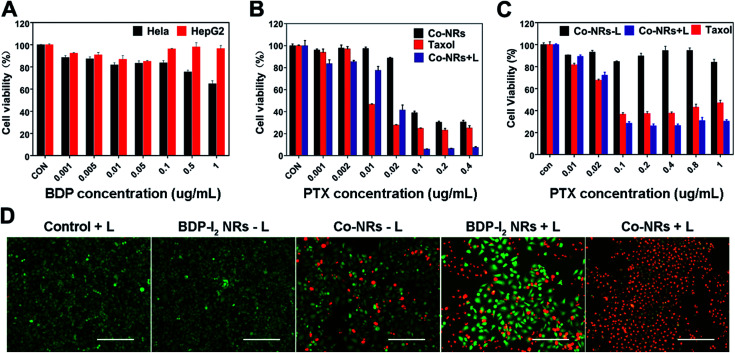
*In vitro* synergistic therapy efficacy of Co-NRs. (A) Relative cell viabilities of HepG2 and HeLa cells incubated with BDP-I_2_ NRs. Cell viabilities of HeLa (B) and HepG2 cells (C) incubated with Co-NPs upon a 540 nm lamp irradiation at 13 W cm^−2^ for 10 min. (D) Fluorescence microscope images of calcein-AM (green, live cells) and propidium iodide (red, dead cells) co-cultured HeLa cells pretreated with various conditions. Scale bars, 40 μm.

The *in vitro* cytotoxicity of Co-NRs against HepG2 and HeLa cells were evaluated by standard thiazolyblue tetrazolium bromide (MTT) assay. As shown in Fig. 4A, BDP-I_2_ NRs exhibited a negligible cytotoxicity without irradiation at the concentration of up to 1 μg mL^−1^ after 48 h incubation, implying the favorable biocompatibility of BDP-I_2_ NRs and excluding its dark cytotoxicity. Under irradiation with a lamp of 540 nm, a dose-dependent cytotoxicity of Co-NRs was observed against both HepG2 and HeLa cells. Co-NPs without irradiation can lead to cell death because of the antitumor effect of PTX. Moreover, under irradiation, Co-NRs exhibited a higher cytotoxicity than that of free PTX (Fig. 4B). The corresponding half-maximal inhibitory concentrations (IC_50_) of Co-NRs without light, free PTX and Co-NRs plus light irradiation against HeLa cells were 0.06, 0.013 and 0.012 μg mL^−1^, respectively. The similar cytotoxicity was also observed against HepG2 cells (Fig. 4C) and the corresponding IC_50_ values of Co-NRs without light, free PTX and Co-NRs plus light irradiation against HepG2 cells were listed in Table S1 (ESI†). Furthermore, the anticancer efficiency of Co-NRs was also examined by the live/dead cell staining test (Fig. 4D). In the control groups, light irradiation alone and BDP-I_2_ NRs in dark almost did not induce cell death. However, light irradiation could enhance the therapeutic effect of Co-NRs *via* photosensitizer-mediated lysosome escape. These results were in accordance with those of MTT.

**Fig. 5:**
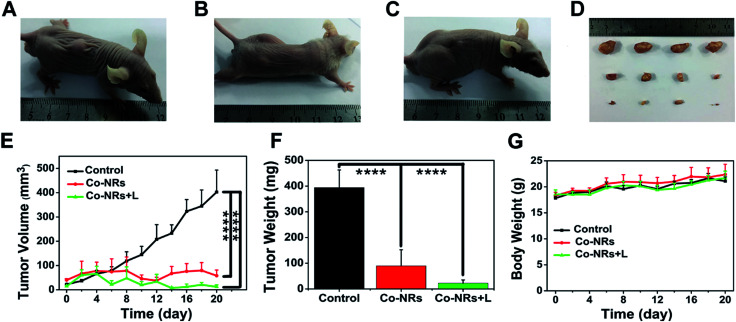
*In vivo* anticancer efficacy. Various groups treated with (A) PBS + light, (B) Co-NRs in dark, (C) Co-NRs + light. (D) Photo of excised tumors. From top to down: PBS + light, Co-NRs in dark and Co-NRs + light group. (E) Changes of tumor volumes of three groups. (F) Tumor weights of three groups. (G) Body weights of mice after different treatments. Statistical significance: ****P < 0.0001.

The therapeutic effect of Co-NRs was further evaluated *in vivo*. Human cervical tumor xenograft (HeLa) in nude mice was utilized as the animal model. The obtained tumor-bearing mice were randomly divided into three groups: PBS plus light irradiation, Co-NRs without light irradiation and Co-NRs plus light irradiation. After intra-tumor injection of Co-NRs at the doses of 1.9 mg kg^−1^, a 450 nm lamp at 200 mW cm^−2^ was employed to irradiate the tumors for 10 min, followed by measuring the tumor volume and body weight. As shown in Fig. 5A–F, PBS plus light irradiation group displayed a sharp tumor growth. In the absence of irradiation, Co-NRs had a relatively satisfied tumor inhibition effect as compared to that of control group due to the favorable therapeutic effect of PTX (Fig. 5B). With the treatment of irradiation, tumors of Co-NRs group were almost completely inhibited (Fig. 5C). After 20 days of observation and measurements, the tumors were harvested from the three groups. Statistical analysis demonstrated that the weights and sizes of tumors treated with Co-NRs were significantly smaller than the control group (Fig. 5E and F). Moreover, as shown in Fig. 5G, the body weights of mice had no significant difference after various treatments, indicating the low side effects and noticeable system toxicity of Co-NRs.

## Experimental section

### Materials and characterization

Milli-Q water was obtained from a Milli-Q system (Millipore, USA). Chemicals and reagents were acquired from commercial sources without further purification. Indocyanine green (ICG) was purchased from Fisher Scientific. Dulbecco's Modified Eagle Medium (DMEM), RPMI-1640 Medium, and fetal bovine serum (FBS) were purchased from Sigma-Aldrich. LysoTracker Red and DAPI were purchased from Life Technologies. Live-Dead Cell Staining Kit was purchased from KeyGen Biotech Co., Ltd. ^1^H NMR spectra were recorded on an Agilent Mercury 400 MHz spectrometer in chloroform-d. Transmission electron microscopy (TEM) images were taken by a JEOL JEM-1011 (Japan) at the accelerating voltage of 100 kV. The fluorescence emission spectra were obtained from a Hitachi fluorometer (F-7500 model). The UV-vis spectroscopy study was performed on a Shimadzu UV-vis spectro-photometer (UV-1800 model). Confocal microscopy imaging was performed using a Zeiss LSM 700 confocal and images were analyzed using ImageJ (NIH).

### Preparation of BDP-I_2_ and paclitaxel (PTX) co-assembly nanorods (Co-NRs)

Co-NRs were prepared by the reprecipitation method. Firstly, the mixture of BDP-I_2_ and PTX were dissolved in acetone (4 mL), then milli-Q water was added dropwise into the solution under stirring. After an overnight stirring, the Co-NRs were obtained and characterized. The BDP-NRs were obtained using the similar method without adding PTX.

### Cell culture

HeLa cells (the human cervical cancer cell line) and HepG2 cells (the human liver hepatocellular carcinoma cell line) were purchased from Jilin University and cultured in culture medium containing DMEM (Dulbecco's modified Eagle's medium), 10% FBS (fetal bovine serum), 100 U mL^−1^ penicillin, and 100 U mL^−1^ streptomycin. The temperature was 37 °C. The concentration of CO_2_ was 5%.

### Biocompatibility of BDP and BDP-NRs *in vitro* by MTT assay

HeLa cells and HepG2 cells were harvested in a logarithmic growth phase and then seeded in 96-well plates at a density of 2 × 10^5^ cells per well. After incubation for 24 h, the medium was replaced with medium containing with drugs, 200 μL per well. Each concentration was set 4 repetitions. Then the light group received irradiation for 10 min, the dark control group received nothing. The green lamp with a wavelength of 540 nm at an intensity of 13 mW cm^−2^ was used to irradiate. After 48 hours, MTT assay was used to measure the cell viability, using the microplate reader and reading at 490 nm.

### Endolysosome escaping and AO staining

HeLa cells were seeded in 6-well plates with cover slips. After incubation for 24 h, the origin medium was replaced by drug-containing medium (BDP-I_2_ at 0.1 μg mL^−1^), 2 hours later, the light group received irradiation for 20 min. Lyso-tracker Red diluted in PBS was added, and the cells were further incubated at 37 °C for 45 min. Then the samples were observed with CLSM.

As for AO staining, the plates without slips were used. After the incubation with drug (BDP-I_2_ at 0.1 μg mL^−1^), light group was treated with irradiation for 20 min. The cells were washed with PBS gently, and then stained with acridine orange (AO, 2.5 mg mL^−1^), and incubated for another 15 min at 37 °C. Fluorescence microscope was employed to observe the images.

### ROS detection

CLSM was used to investigate the generation of intracellular ROS. HeLa cells obtained from the logarithmic growth phase were seeded in 6-well plate with cleaned cover slip. After an incubation of 24 h, we switched the culture medium with medium with drugs or not. The final BDP-I_2_ concentration was 0.1 μg mL^−1^. Two hours later, the light group received an irradiation of 20 min. The cells were washed immediately with DMEM solution without FBS, and then, the DMEM solution containing DCFH-DA was added as a sensor. The cover slips were observed with CLSM as soon as possible after further incubation for 45 min at 37 °C. The whole process was carried out dark in case of the quenching. The excitation wavelength of CLSM was 488 nm, and the emission band pass was 500–550 nm.

Furthermore, ICG and UV-vis spectroscopy were employed to evaluate the singlet oxygen generation ability *in vitro*. ICG water solution (1 mg mL^−1^) was prepared at first. Then 21 μL of the solution was added into 3 mL of water and 3 mL of Co-NRs (BDP-I_2_ concentration at 5 μg mL^−1^), separately. Both samples received an irradiation of 240 s, 30 s per time, and were detected 9 times. The changes in 779 nm were analyzed.

### Animal study

Male nude mice were purchased from Jilin University, China (56–84 d, 15–20 g) and maintained under control. All the experiments were performed in strict accordance with the NIH guidelines for the care and use of laboratory animals (NIH Publication No. 85-23 Rev. 1985) and was approved by the guidelines of the Committee on Animal Use and Care of Chinese Academy of Sciences. Hela cells (100 μL) were injected into the back of nude mice. One week later, 200 μL of drugs were intratumoral injected into the tumors. The control group received PBS instead. Two hours later, the control and light group received an irradiation of 450 nm, 200 mW cm^−2^, for 10 min. In the next 20 days, body weight and tumor volume of mice were measured every two days. Finally, the tumors were harvested and weighed.

## Conclusions

In summary, a nanotheranostic agent was successfully prepared by the co-assembling of photosensitizer BDP-I_2_ and chemical anticancer drug PTX. The obtained Co-NRs could effectively be internalized by cells and enhance the lysosome escape of PTX, promote the cell cytotoxicity *in vitro*. *In vivo* experiments further validated the excellent therapeutic efficacy of as-prepared Co-NRs. This present work emphasizes the progress in the field of small molecule self-assembly and helps to elevate these to later clinical trials.

## Conflicts of interest

There are no conflicts to declare.

## Supplementary Material

RA-008-C8RA00067K-s001
