# Supplementary material for: Self-assembled organic nanorods for dual chemo-photodynamic therapies
Source: RSC Adv. 2018 Feb 1;8(10):5493–9. doi: 10.1039/c8ra00067k (PMC9078097; doi:10.1039/c8ra00067k)
Supplement: RA-008-C8RA00067K-s001 [file RA-008-C8RA00067K-s001.pdf]

Electronic Supplementary Material (ESI) for RSC Advances.
This journal is © The Royal Society of Chemistry 2018

Electronic Supplementary Information (ESI)

Self-assembled organic nanorods for dual chemo-photodynamic Therapies

Yuanyuan Li,[a] Xiuli Hu,[b] Xiaohua Zheng,[c] Yang Liu,[d] Shi Liu,[b] Ying Yue,*[a] Zhigang Xie[b]*

a. The First Hospital of Jilin University, Xinmin Street, Changchun, Jilin 130021, PR China. E-mail: yying119@126.com

b.Applied Chemistry, Chinese Academy of Sciences, Changchun 130022 (China). E-mail: lily@ciac.ac.cn

c.University of Science and Technology of China, Hefei 230026, PR China.

d.Department of Chemistry, Northeast Normal University,5268 Renmin Street, Changchun 130024, P. R. China.se Academy of Sciences, Changchun 130022 (China).

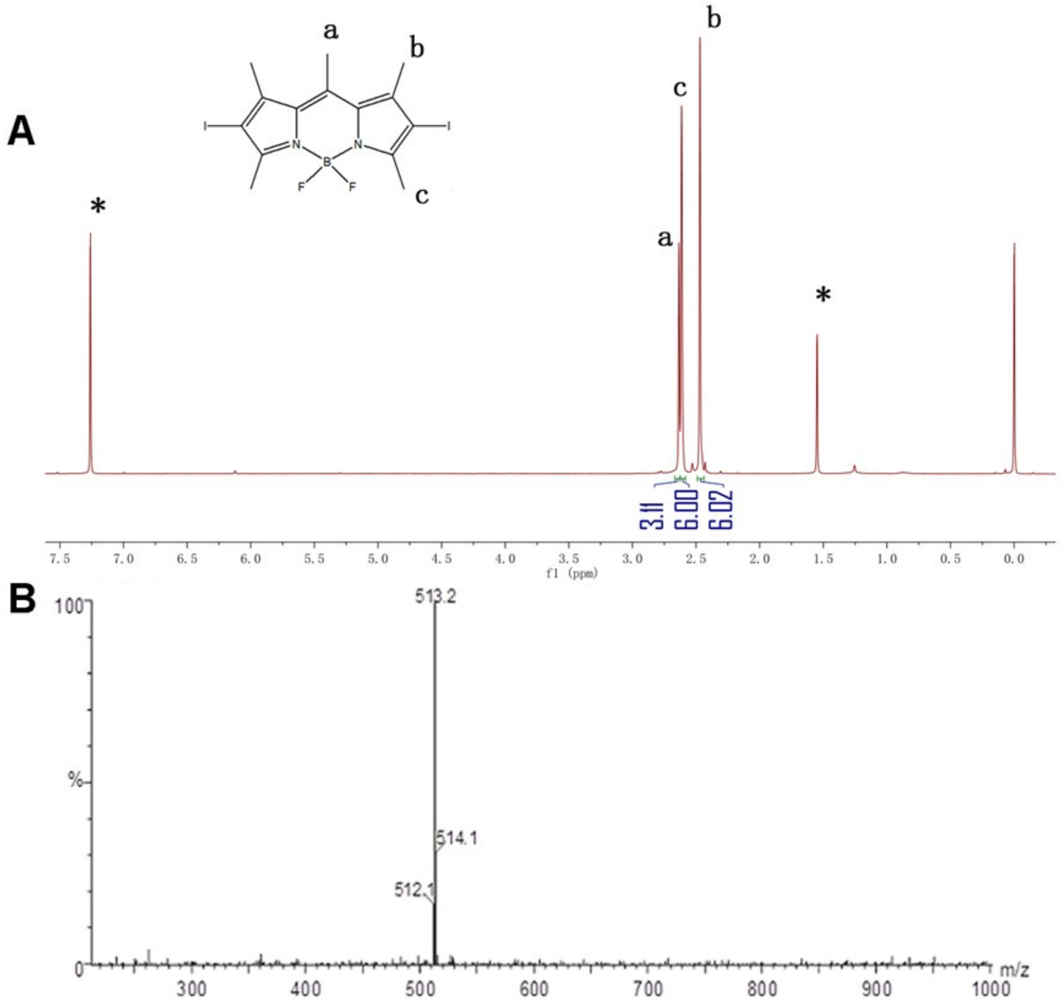

Figure S1. (A) ^{1}H NMR spectra of BDP-I_2. (B) MALDI-TOF mass spectra of BDP-I_2.

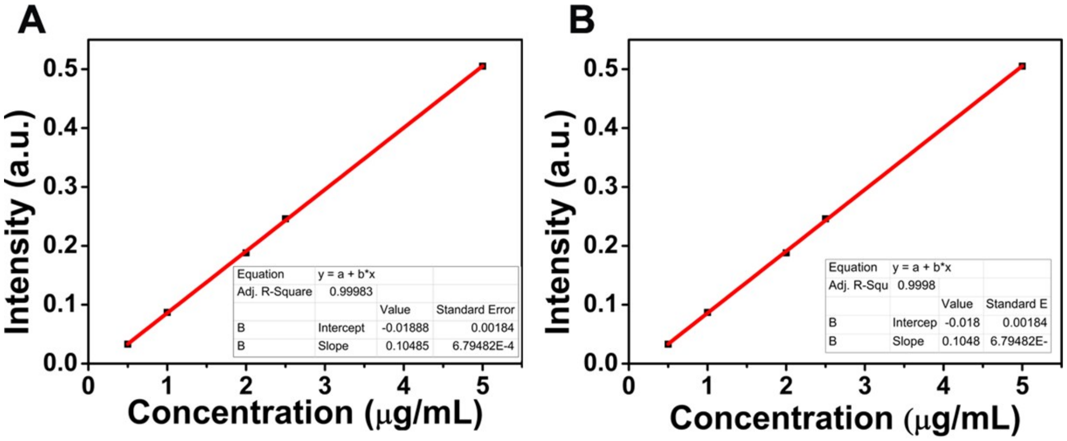

Figure S2. (A) Standard curve of BDP-I_2 in DMF and water (9:1). (B) The standard curve of high performance liquid chromatography (HPLC) result of PTX.

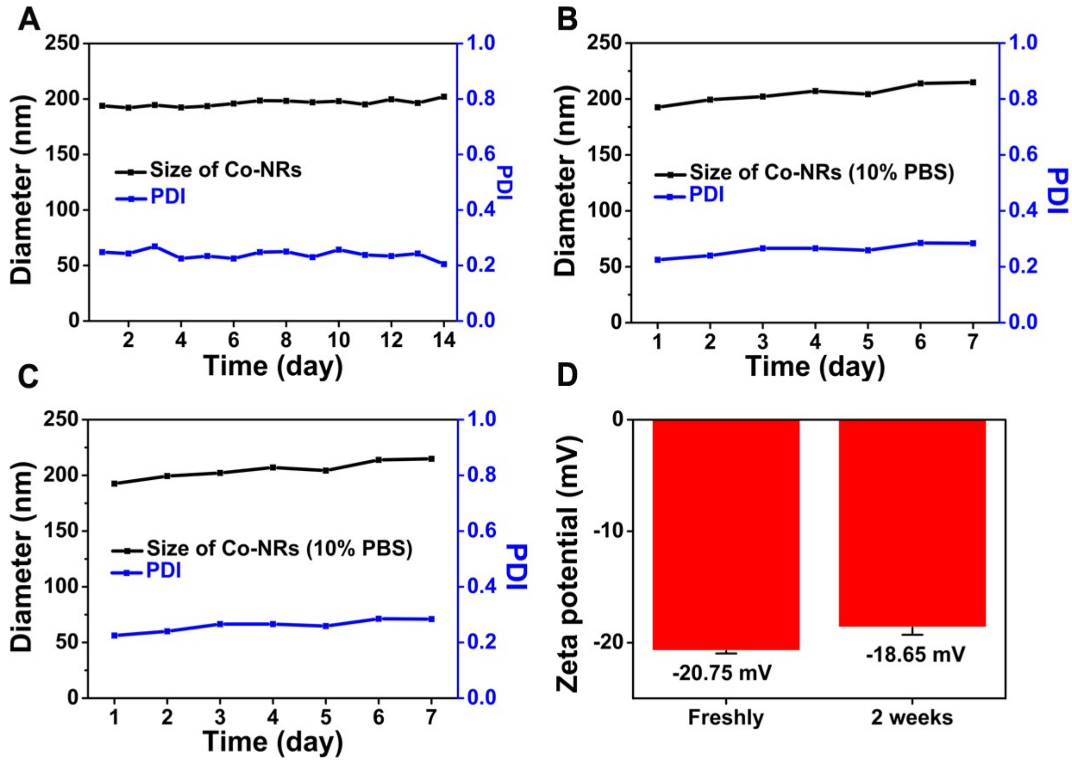

Figure S3. The physiological stability of Co-NRs in water (A) and 10% FBS (B), 10% PBS (C) for 14 or 7 days. (D) The Zeta potential change of Co-NRs after 14 days.

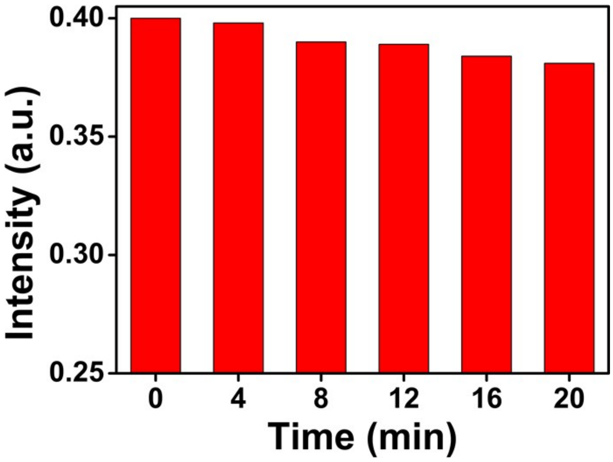

Figure S4. The UV-Vis absorption intensity changes of Co-NRs under a 540 nm lamp at an intensity of 13 mW cm^{-2}.

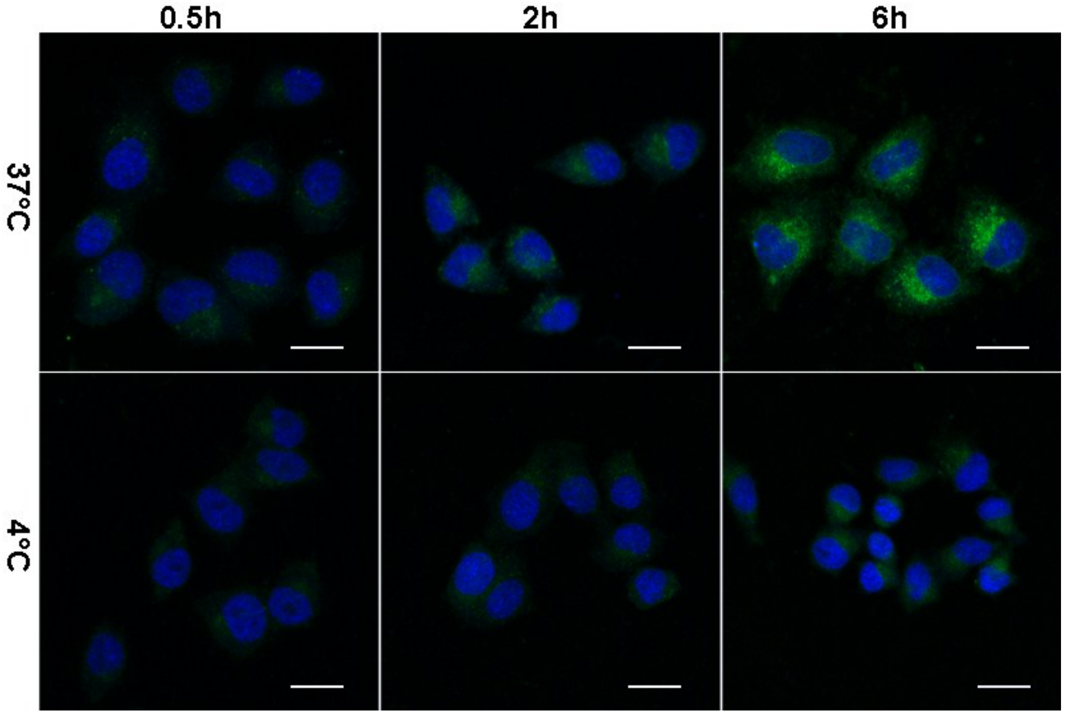

Figure S5. Representative CLSM images of HeLa cells incubated with Co-NRs for 0.5, 2, 6 h at 4 ^{o}C and 37 ^{o}C, respectively. Scale bars, 20 μm.

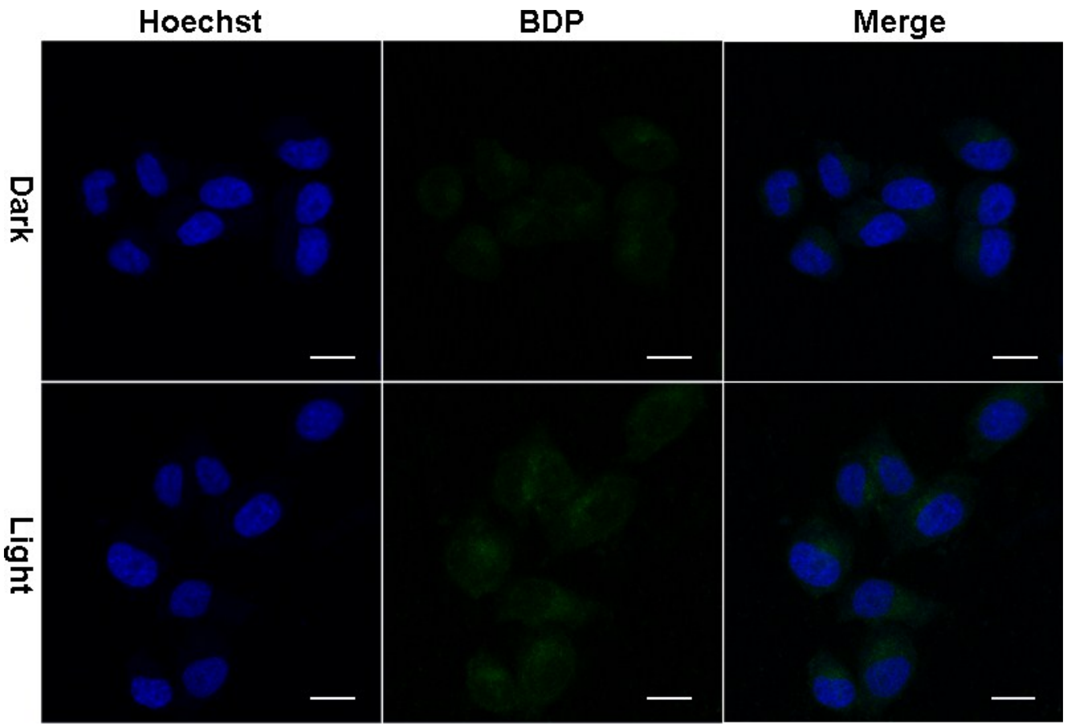

Figure S6. Representative CLSM images of HeLa cells incubated with Co-NRs with and without irradiation, respectively. Scale bars, 20 μm.

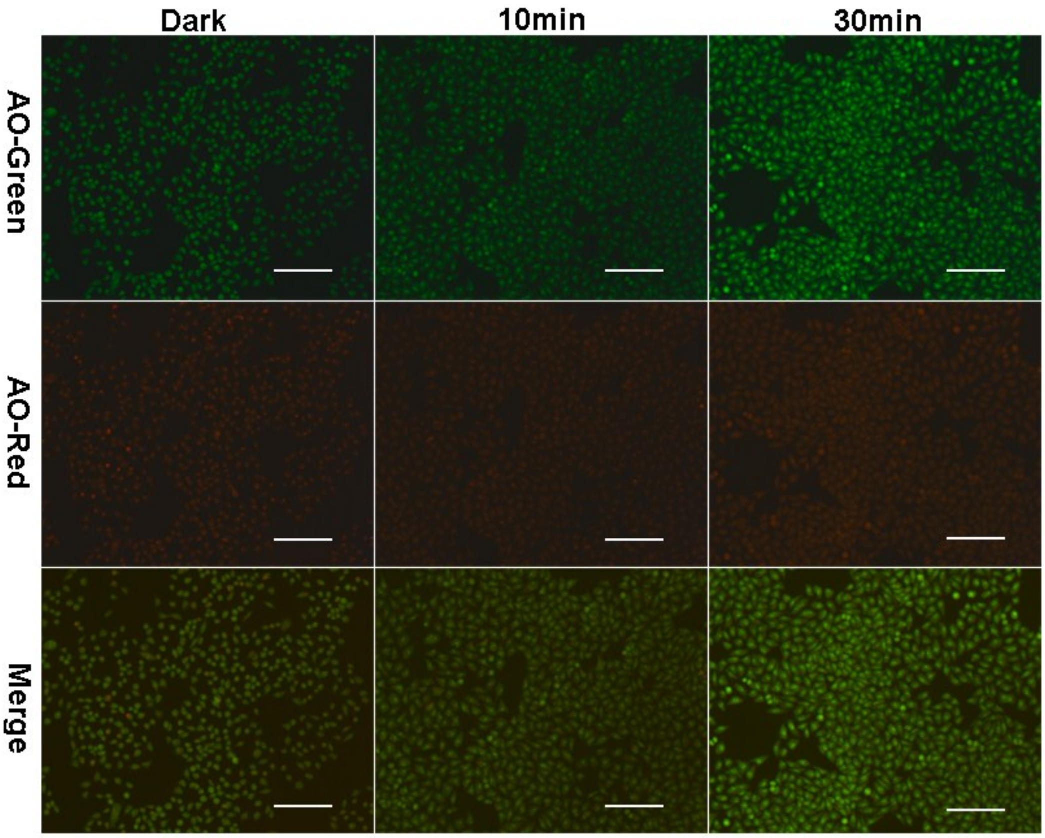

Figure S7. Effects of Co-NRs with and without irradiation on AO staining of HeLa cells. From the top to bottom: green fluorescence of AO excited by 488 nm channel, red fluorescence of AO excited by 555 nm channel and merged results. Scale bars: 50 μm.

Table S1. IC_{50} values of Co-NRs against HeLa and HepG2 Cells.

IC_{50} (μg/mL)	Co-NRs	Taxol	Co-NRs + L
HeLa	0.06	0.013	0.012
HepG2	--	0.081	0.064
